# Deterministic Areal Enhancement of Interlayer Exciton Emission by a Plasmonic Lattice on Mirror

**DOI:** 10.1021/acsnano.4c00061

**Published:** 2024-05-14

**Authors:** Jiasen Zhu, Fuhuan Shen, Zefeng Chen, Feihong Liu, Shuaiyu Jin, Dangyuan Lei, Jianbin Xu

**Affiliations:** † Department of Electronic Engineering, The Chinese University of Hong Kong, Shatin 999077, Hong Kong SAR, China; ‡ School of Optoelectronic Science and Engineering and Collaborative Innovation Center of Suzhou Nano Science and Technology, Soochow University, Suzhou 215006, China; § Department of Materials Science and Engineering, City University of Hong Kong, Kowloon 999077, Hong Kong SAR, China

**Keywords:** plasmonics, photoluminescence, interlayer exciton, out-of-plane dipole, localized surface plasmon resonance

## Abstract

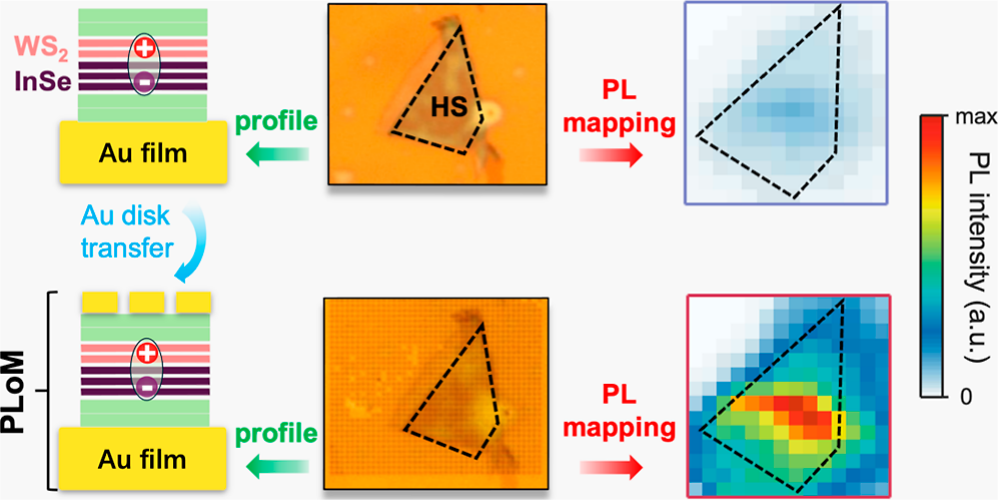

The emergence of interlayer excitons (IX) in atomically thin heterostructures of transition metal dichalcogenides (TMDCs) has drawn great attention due to their unique and exotic optical and optoelectronic properties. Because of the spatially indirect nature of IX, its oscillator strength is 2 orders of magnitude smaller than that of the intralayer excitons, resulting in a relatively low photoluminescence (PL) efficiency. Here, we achieve the PL enhancement of IX by more than 2 orders of magnitude across the entire heterostructure area with a plasmonic lattice on mirror (PLoM) structure. The significant PL enhancement mainly arises from resonant coupling between the amplified electric field strength within the PLoM gap and the out-of-plane dipole moment of IX excitons, increasing the emission efficiency by a factor of around 47.5 through the Purcell effect. This mechanism is further verified by detuning the PLoM resonance frequency with respect to the IX emission energy, which is consistent with our theoretical model. Moreover, our simulation results reveal that the PLoM structure greatly alters the far-field radiation of the IX excitons preferentially to the surface normal direction, which increases the collection efficiency by a factor of around 10. Our work provides a reliable and universal method to enhance and manipulate the emission properties of the out-of-plane excitons in a deterministic way and holds great promise for boosting the development of photoelectronic devices based on the IX excitons.

## Introduction

Two-dimensional (2D) heterostructures consisting of different layered materials offer a versatile platform for ultrathin electronic and photoelectronic devices,^1,2^ as well as for frontier scientific research, such as superconducting and quantum computing. Among them, the heterostructures with transition metal dichalcogenide (TMDC) materials recently attracted great attention due to their photoelectronic performance and unique physical properties.^3,4^ The combination of two different 2D TMDC layers hosts the atomically sharp type-II electronic interlayer, resulting in the formation of lower-energy interlayer excitons with electrons and holes residing in different material layers.^5,6^ Consequently, the spatially separated charges from IX give rise to the out-of-plane (OP) dipole moment.^7,8^ In some previous reports, the bilayer TMDC heterostructures composed of monolayer TMDC with K-valley IX have been studied, which necessitated the precise alignment of the relative rotation angle of the two TMDC lattices.^9^ Instead, the pioneering work^10^ by aligning bilayer (2L) WS_2_ and multilayer (multi-L) InSe has successfully demonstrated the direct interlayer transition at the Γ point in the momentum space, circumventing the momentum mismatch issues in the experiment.

Typically, compared with the intralayer exciton in multi-L TMDC, IX emission is quite weak because of the extremely low oscillator strength (10^–2^ of that of intralayer exciton), greatly hindering the development of photoelectronic devices based on TMDC heterostructures.^11^ It is also well known that the emission properties of emitters are also determined by the surrounding environment.^12−16^ Some efforts, such as using dielectric photonic crystal, have successfully manipulated and enhanced the IX emission.^17−20^ Nevertheless, because of the high-quality factor (or *Q* factor) of the dielectric cavity, the dielectric cavity resonance cannot overlap with the broad spectra from IX.^21^

On the other hand, the plasmonic particle-on-mirror (PoM) structure, showing spectrally broad plasmonic resonance, has been applied to enhance emissions of IX^22^ and the dark excitons in monolayer WSe_2_ with the highly confined gap mode.^18,23,24^ To form PoM, the material is transferred onto the metal surface, separated by a dielectric layer, and the particles are spin-coated on the material surface. As a result, the location and orientation (if it is not a perfect sphere) of the nanoparticle on the material are highly random, making deterministic emission enhancement challenging in experiment.^25,26^

In this report, we extend the PoM structure to the plasmonic-lattice-on-mirror (PLoM) structure, where the plasmonic lattice (disk array) is placed on a gold film (mirror) to achieve the deterministic and areal enhancement of IX emission from the WS_2_/InSe heterostructure (HS). The PLoM structure shows both the surface propagating plasmon (PSP) and localized surface plasmon (LSP) modes. The near-field profile of the LSP mode demonstrates pronounced electric field enhancement in the OP direction, highly aligned with the IX dipole momentum. By inserting the HS into the PLoM, we achieve a pronounced emission enhancement of up to 350. In addition, the enhancement is homogeneous across the entire HS region and can be controlled by altering the LSP resonance. Our work is promising to improve the performance of optoelectronic devices consisting of IX, such as infrared photodetector,^27^ light-emitting diodes^11,28^ and photovoltaics.^2,8^

## Results and Discussion

**Figure 1:**
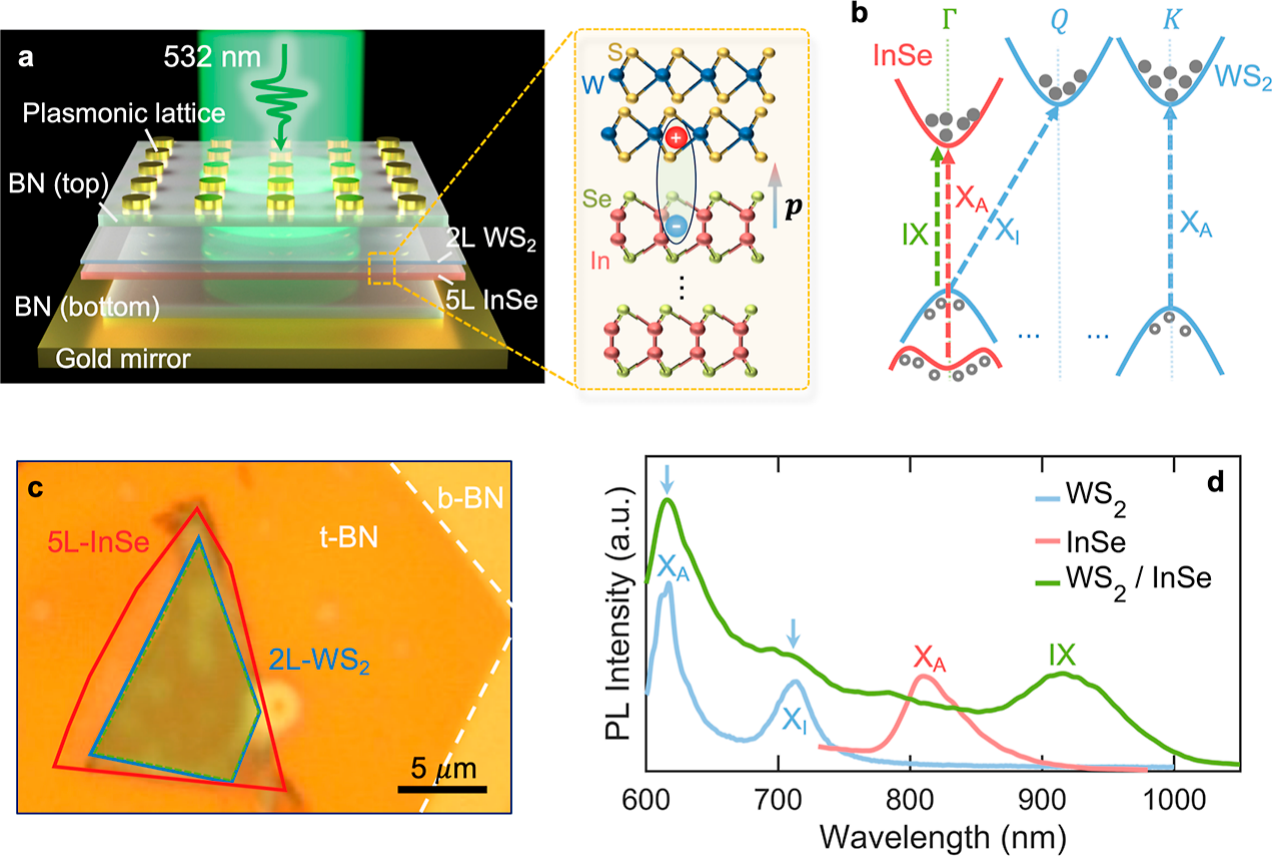
Design of a plasmon-enhanced IX system and optical properties of IX. (a) Schematic diagram of a WS_2_/InSe HS inserted into a PLoM structure. The right inset sketches the formation of IX in the WS_2_/InSe HS, where *p* represents the IX dipole moment aligned in the OP direction. Atoms: sulfur in gold, tungsten in blue, indium in pink, and selenium in olive green). (b) Schematic band alignment of the WS_2_/InSe HS: the blue and pink lines represent the conduction and valence band edges of 2L-WS_2_ and of 5L-InSe, respectively. The blue and pink dash arrows indicate the intralayer transitions of the individual WS_2_ (direct X_A_ and indirect X_I_) and InSe (direct X_A_) layers, respectively, while the green dash arrow represents the interlayer transition of the WS_2_/InSe HS (IX). (c) Micrograph of the WS_2_/InSe HS encapsulated with top and bottom BN. Individual 2D flakes are outlined for clarity: WS_2_ (blue dashed lines), InSe (red dashed lines), and BN (white dashed lines). (d) Measured PL spectra of individual 2L-WS_2_ (blue), 5L-InSe (pink), and the WS_2_/InSe HS (green) at 77 K.

Figure 1a shows a schematic diagram of our designed structure, where the WS_2_–InSe HS is sandwiched between the gold plasmonic lattice and the gold film. The HS is composed of 2L of WS_2_ and 5L of InSe, encapsulated by the top and bottom BN (total thickness of 25–30 nm) to isolate air and moisture from the environment. All the constituent materials are obtained by the mechanical exfoliation method. WS_2_ and InSe with the designed atomic layer number are selected by the optical microscope image contrast and confirmed with PL spectra. The thickness of BN is measured by an atomic force microscope (AFM). The encapsulated HS was transferred onto the gold film (thickness = 50 nm) for optical characterization and further processing, whose optical microscopic image is shown in Figure 1c. As indicated in the inset in Figure 1a, the IX of the WS_2_/InSe heterostructure is formed through the Coulomb interaction between a hole from the InSe layer and an electron from the WS_2_ layer, having a permanent dipole moment *p* perpendicular to the heterostructure interface, i.e., aligned in the OP direction.

We first characterize the emission properties of HS and its constituent materials to confirm the existence of the IX. Figure 1d shows the measured PL spectra at 77 K from the individual WS_2_ layer (outlined in blue in Figure 1c), the InSe layer (outlined in red), and their HS region (highlighted in green). Two peaks from WS_2_ originate from the direct intralayer transition at the *K* point (X_A_ ∼ 618 nm) and the indirect intralayer transition (X_I_ ∼ 710 nm) between electrons at the *Q* point and holes at the Γ point.^10^ For InSe, only one prominent peak due to a direct *A* exciton transition is observed at around 815 nm within our measured spectral range. The PL spectrum from HS, in addition to the corresponding signature peaks from constituent layers (marked by blue arrows), exhibits a pronounced peak located at a lower energy (∼905 nm, i.e., 1.34 eV). This low-energy peak becomes dominant on cooling (see temperature evolution of the PL spectra of the HS, WS_2_, and InSe layers in Figure S1). Our measured results are in great agreement with the previous report^29^ on the same WS_2_/InSe HS and therefore confirm the interlayer transition for this low-energy PL peak.

**Figure 2:**
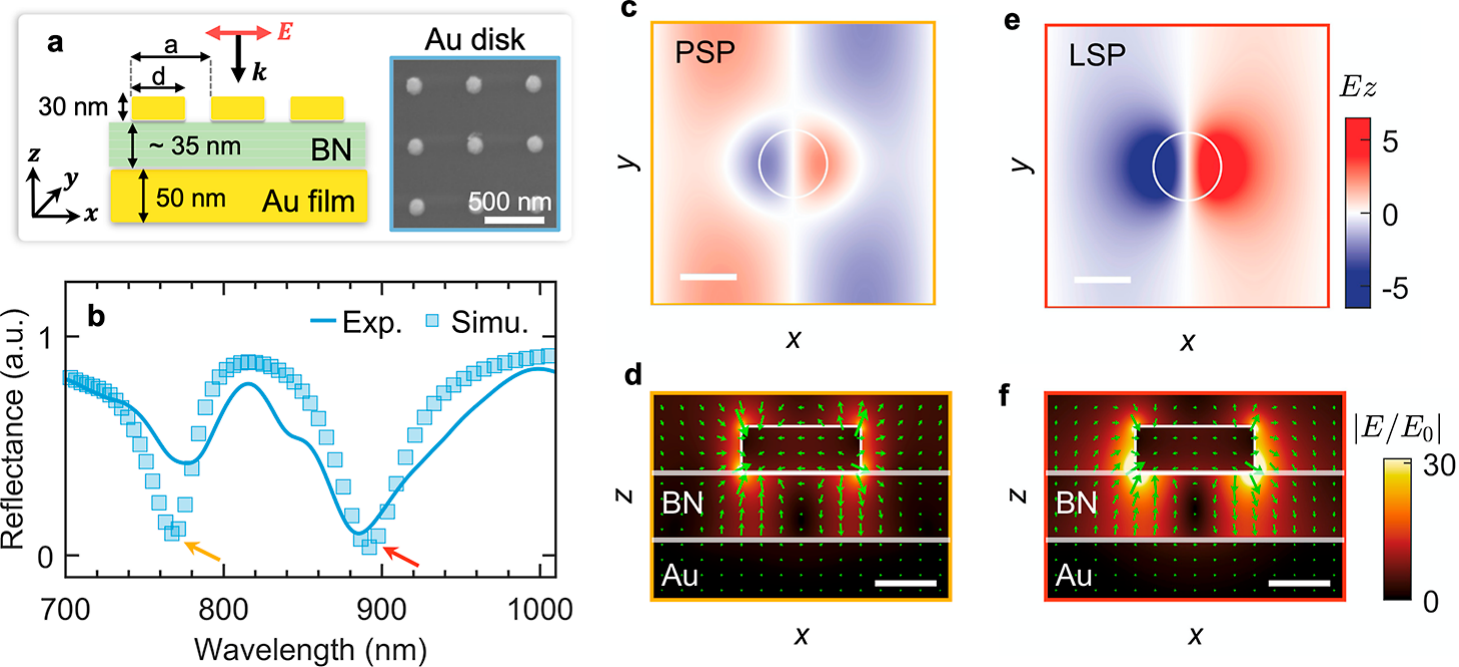
Plasmonic properties of a PLoM structure. (a) Left panel: side view (*xz*-plane) of the PLoM structure used in simulation. *d* is the diameter of an individual gold disk, and *a* is the pitch of the array. Right panel: SEM image of one representative gold disk array (scale bar: 500 nm). (b) Measured (blue curve) and simulated (blue square) reflectance spectra of the PLoM structure shown in (a) under normal incidence. (c,e) Simulated distribution profiles of the *z*-component electric field right above the top surface of the gold film at the PSP (c) and LSP (e) resonance wavelengths (scale bar: 100 nm). (d,f) Simulated electric field distribution profile in the *xz*-plane at PSP (d) and LSP (f) resonance wavelengths (scale bar: 50 nm). In the simulation, *d* = 110 nm and *a* = 500 nm. The green arrows represent the real part of vectorial electric field projected in the *xz*-plane.

The left panel in Figure 2a shows the schematic *x*–*z* profile of the bare PLoM (i.e., without HS), where the Au disk arrays are placed on the surface of the BN/Au film. The thickness of the BN is around 35 nm, which matches the total thickness of the top and bottom BNs of the HS (Figure 1c). The Au disk arrays with various diameters *d* and pitches *a* (see Figure S2) were fabricated via sequential electron beam lithography, electron-beam evaporation, and lift-off procedures.^15,30−32^ The scanning electron microscopy (SEM) image of a representative array is shown in the right panel of Figure 2a. To place the disk arrays onto the target area (e.g., BN flake or HS), the disk array was first spin-coated with a layer of PMMA (thickness of ∼200 nm) and then transferred with a standard wet transfer procedure, which is widely applied in transferring 2D materials (more details about transfer procedures can be seen in Figure S3).

The optical properties of the PLoM structure were characterized by measuring the reflectance spectrum under a normal incident broadband (700 to 1000 nm) linearly polarized plane wave. As Figure 2b shows, two dips are revealed in the spectrum, which can be well reproduced by the finite-difference time-domain (FDTD) simulation using similar geometric parameters as used for the fabricated PLoM structure. The dip at around 765 nm corresponds to the propagating surface plasmon (PSP) bound at the interface between the gold and dielectric material (i.e., BN). For the excitation of this PSP mode, the Au disk array serves as the two-dimensional grating,^33^ providing an additional momentum  to couple the incident wave to the metal surface. *p* and *q* represent the diffraction orders. The near-field (*z* component of the electric field, i.e., *E*_*z*_) distribution on the top surface of the gold film (Figure 2c) at 765 nm shows the propagating instinct of this mode.

In comparison, as indicated in Figure 2e, the near-field (*E*_*z*_) distribution for the lower-energy mode (895 nm) is more enhanced and localized around the projected edge (marked by the white circle) of the nanodisk on the *xy*-plane. This corresponds to the localized surface plasmon (LSP) resonance.^12^ Due to the interaction between the disk and its image (the gold film serves as the mirror),^34^ the electric field of the LSP mode is greatly enhanced within the gap, and the electric field is majorly aligned in the OP or *z* direction,^35,36^ as Figure 2f shows. Compared with the LSP, the PSP mode shows a relatively weak electric field enhancement within the gap (Figure 2e). In addition, we also measured and simulated the reflectance spectra for the disk arrays with various diameters and pitches, as exhibited in Figures S4 and S5, to show the different parameter-dependent characteristics of PSP and LSP modes. As Figure S5a shows, the PSP mode is invariant with the disk diameter, while the LSP shows an unambiguous red shift with the diameter increased. On the other hand, once the pitch is increased, as Figure S5b exhibits, PSP shows a significant red shift, while LSP is only slightly red-shifted due to the interaction between PSP and LSP modes.

**Figure 3:**
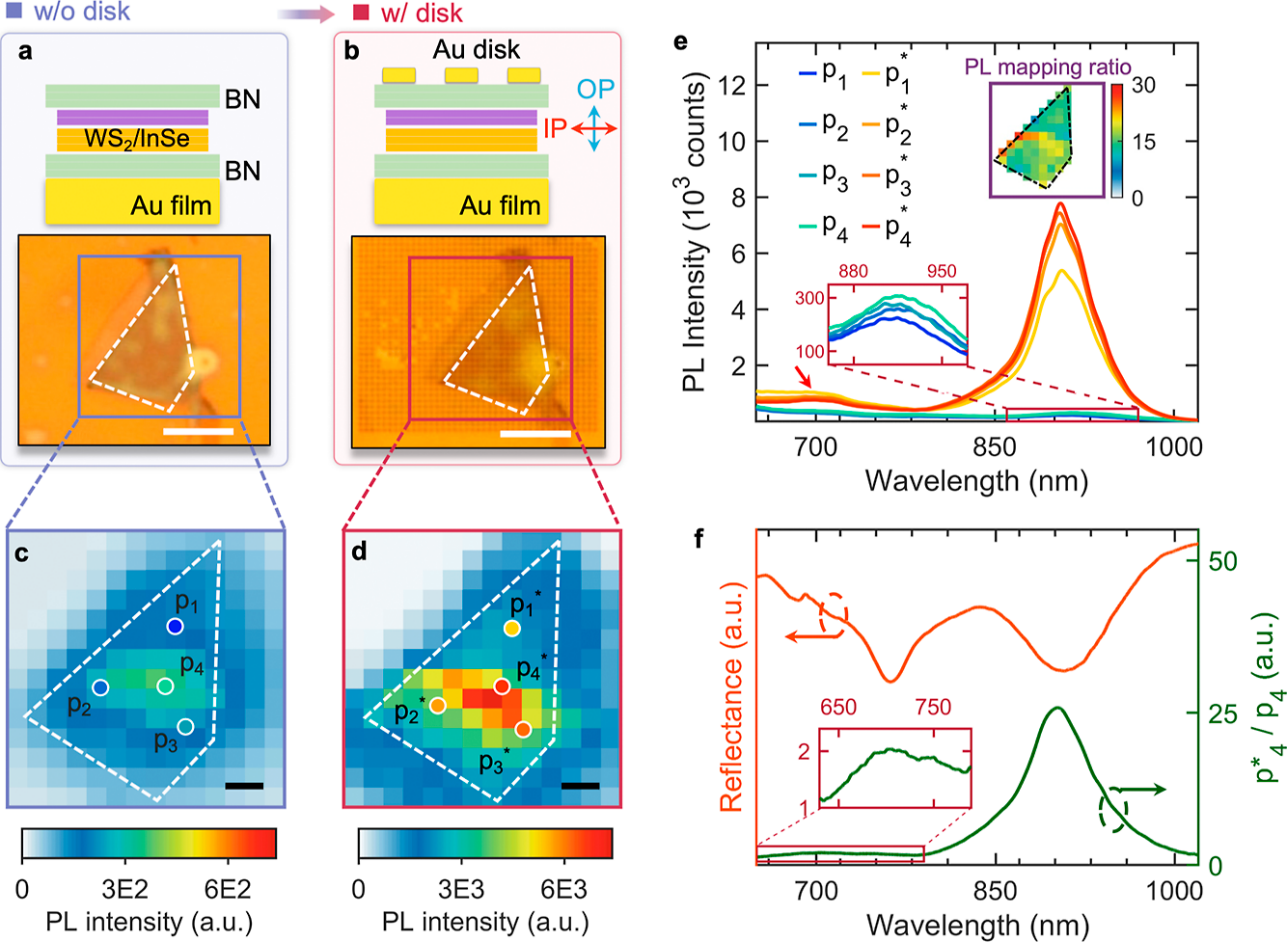
PL enhancement of IX by the PLoM structure. (a) Schematic structure of a BN-encapsulated HS on a gold thin film (top) and its corresponding optical image (bottom). (b) Same as (a), but with an array of Au nanodisks transferred onto the BN-encapsulated HS. The scale bars in both optical images are 5 μm. (c,d) PL mapping images for the HS without (c) and with (d) the gold nanodisk array. The mapping step size is 1 μm and the scale bars in both images are 2 μm. (e) PL spectra from four selected locations labeled, respectively, in (c,d). Bottom-left inset shows the zoomed-in PL spectra highlighted in the red box, and the upper-right inset is the PL intensity ratio profile between Figure 3d and 3c. (f) Reflectance spectrum (red) of the PLoM structure (with HS inserted) and the calculated intensity ratio between the PL spectra collected at points p_4_* and p_4_ (green). The inset shows the zoom-in spectrum highlighted in the red box.

Next, using the same wet transfer method, we transferred the disk arrays onto the WS_2_/InSe HS, as Figure 3a,b exhibits. After transfer, the disk arrays were well retained on the surface of the top BN (as the optical microscope image in Figure 3d shows). We performed the PL mapping for the same HS without and with the disk arrays on top at 77 K (if not otherwise noted, the following PL measurements were all conducted at 77 K). The excitation wavelength was 532 nm, and PL signals were collected by an optical microscope with a numerical aperture NA = 0.5. As Figure 3c shows, the emission of original HS is not uniform over the whole HS region, which is possibly due to the various interface contacts between WS_2_ and InSe when they were assembled. After the disk array was transferred onto the HS (Figure 3b), the PL emission of the HS/PLoM hybrid structure exhibits a significant increase of more than 1 order of magnitude. Meanwhile, a similar inhomogeneous emission intensity distribution in Figure 3c can also be observed in Figure 3d when the disk array is on top.

Four representative locations (p_1_ to p_4_) are selected from the HS region, as indicated in Figure 3c,d, and their PL spectra are shown in Figure 3e correspondingly. For the original HS (i.e., without disk arrays), locations p_1_, p_2_, p_3_, and p_4_ have increasingly stronger IX emission intensities, i.e., IX PL emission: p_4_ > p_3_ > p_2_ > p_1_, which is more clearly shown in the zoom-in spectra in the bottom-left inset in Figure 3e. When the HS is inserted in the PLoM, IX emission intensities for the same four locations show pronounced enhancements (IX PL emission: p_4_* > p_3_* > p_2_* > p_1_*). In addition, an asymmetric and broad peak (noted by a /red arrow), due to the PSP mode, emerged within the range of 700 to 750 nm for p_1_*–p_4_* measurements. To show the enhancement of IX emission due to the PLoM structure, the PL enhancement p_n_*/p_*n*_ (with *n* from 1 to 4) is calculated, as shown in Figure S6. In all locations, the PL enhancement of 20- to 25-fold is shown. Besides that, the mapping data of the ratio between Figure 3d and 3c are exhibited in the upper-right inset in Figure 3e, showing the relatively homogeneous IX emission enhancement of 15- to 25- fold across the whole HS region.

The calculated p_4_*/p_4_ value is compared with the measured reflectance spectrum of PLoM (with HS inserted), as indicated in Figure 3f. Compared with that of the bare PLoM in Figure 2b, the reflectance spectrum (orange curve) shows a slight modification due to the existence of HS. For the LSP mode (∼890 nm), because of the enhancement of the electric field in the OP direction within the PLoM dielectric gap (Figure 2f), it could efficiently couple with the interlayer transition dipole in the same direction and therefore result in a 25-fold emission enhancement. While for the PSP mode (∼760 nm), which is close to the indirect transition of X_I_ (see Figure 1d) of WS_2_ in the spectrum, it only slightly enhances the emission of X_I_ (the zoom-in inset in Figure 3f) due to the reduced electric field (Figure 2d) and significant energy mismatch (Figure 3f).

Here, the average PL enhancement factor can be calculated by
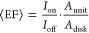
1where *I*_on_ (*I*_off_) represents the integration of the measured PL from the disk-coupled (uncoupled) HS over the whole HS area (i.e., within the white dashed boundary in Figure 3c,d). *A*_unit_ = *a*^2^ is the unit area of disk, and *A*_disk_ = π*r*^2^ is the disk area (see Figure S7). According to the above definition, the emission enhancement factor ⟨EF⟩ for the sample (*a* = 500 nm, *d* = 120 nm), as shown in Figure 3a,b, can be estimated at around 350. The total enhancement factor EF majorly originates from three parts,^37^ i.e.,
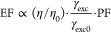
2

**Figure 4:**
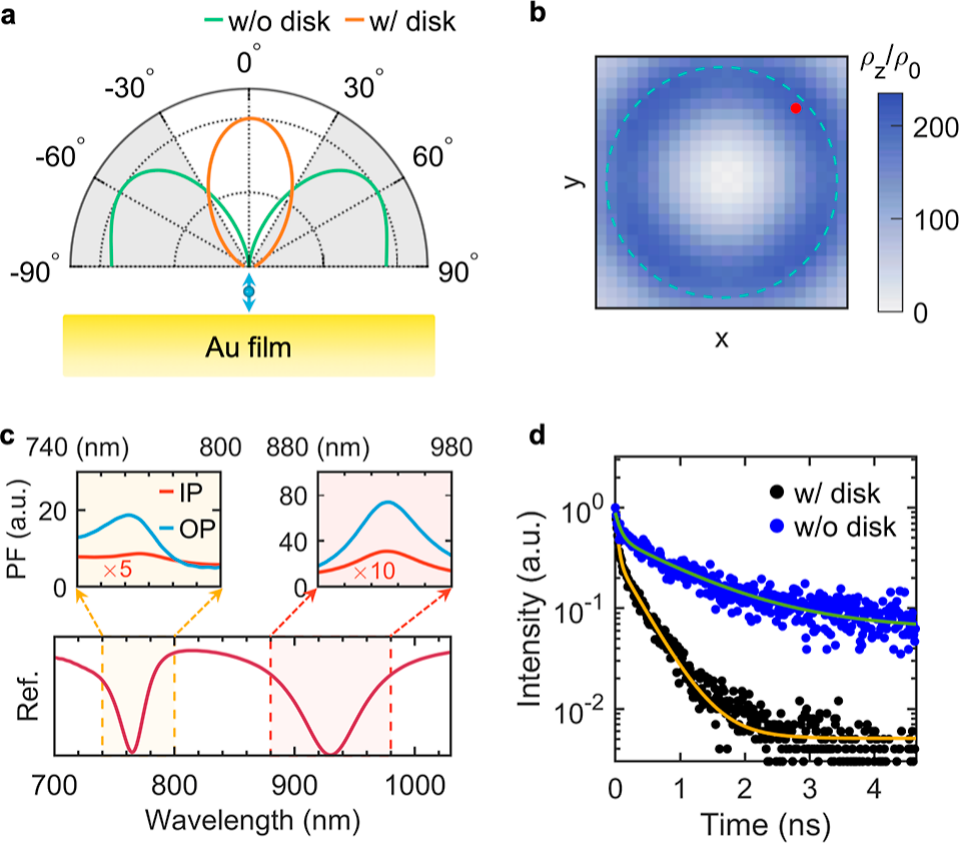
Physical origins of the IX PL enhancement by the PLoM structure. (a) Simulated far-field radiation patterns for an OP dipole without (as indicated in the top panel of Figure 3a) and with (as indicated in the top panel of Figure 3b) an array of gold nanodisks on the top. (b) Spatial distribution of the OP LDOSs in the middle *xy*-plane of the PLoM gap. (c) Calculated PF for in-plane (short for “IP”, red curve) and out-of-plane (short for “OP”, blue curve) dipoles in the LSP (upper-left corner) and PSP (lower-right corner) resonance bands, respectively. (d) Measured PL lifetimes of IX from the HS without (blue dots) and with (black dots) the array of gold nanodisks on top. The blue and orange represent biexponential fitting of the experimental data.

The (η/η_0_) term represents the improved collection efficiency due to the nanoantenna, where η (η_0_) is the collection efficiency with (without) the nanodisk above the emitter. Our FDTD simulation results in Figure 4a show that the radiation pattern of bare IX dipole (without disk) exhibits two symmetric lobs with respect to the surface-normal direction, resulting in a relatively low collection efficiency from the top side. The PLoM, serving as the patch antenna array, greatly modifies the radiation pattern into a single lob orienting along the surface-normal direction. The objective with NA = 0.5 adopted in our measurements is estimated to achieve η/η_0_ = 10 collection efficiency enhancement due to the nanodisk (see the analysis in Table S1). Furthermore, IX emissions collected by objectives with different NAs (NA = 0.65 and NA = 0.8) are also measured, as shown in Figure S13. With the NA of objective increased in the measurements, the term (η/η_0_) shows an unambiguous decrease, which is consistent with the simulation results (Table S1). This tendency can be intuitively understood from the far-field radiation in Figure 4a. An objective with a larger NA (i.e., larger collection angle) would greatly increase the collection efficiency of the OP dipole without disk (green curve in Figure 4a), while having a limited influence on the disk-modified radiation field by the dipole (orange curve in Figure 4a).

The second term  represents the excitation enhancement due to the PLoM,^38^ where γ_exc_(γ_exc0_) is the excitation rate with (without) the nanodisk. However, because of the absence of the resonance at the excitation wavelength for our PLoM structure, the electric field shows a negligible enhancement at 532 nm (Figure S8), and hence, the effects of this term can be ignored.

The third term Purcell factor PF, defined as , represents the spontaneous emission rate enhancement of an emitter due to the change of photonic environment,^39,40^ where γ_sp_(γ_sp0_) represents the spontaneous decay rate of the emitter coupled (uncoupled) to the antenna, and *t*_sp_(*t*_sp0_) represents the corresponding radiative lifetime. In general, the spontaneous emission rate of a dipole is given by^37^
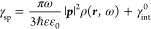
3where ε and ε_0_ are the relative dielectric constant of the environment and the vacuum dielectric constant, respectively, ω is the emission frequency, *r* is the position, *p* is the dipole moment of the emitter, γ_int_^0^ is the internal nonradiative decay rate of the emitter, and ρ(***r***,ω) represents the local density of states (LDOSs), which is enhanced by the large electric field in the PLoM gap
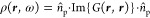
4where *n̂*_p_ is the orientation of the transition dipole of the emitter (i.e., the OP direction or the *z*-direction in this system), and *G*(***r*,*r***) is the dyadic Green’s function, representing the interaction between the electric field and the emitter due to its own radiation field.

LDOSs are calculated at the LSP mode resonance (i.e., 890 nm) through inserting an OP dipole (as the blue double arrow indicates in Figure 3a) at the middle of the BN layer in the PLoM structure, as shown in Figure 2a (left panel). As Figure 4b shows, the LDOSs are greatly enhanced within the gap of the PLoM structure. The enhancement is highly dependent on the position of the dipole, where the LDOSs show a dramatic enhancement of more than 200 at around the projected edge (blue dashed curve) of the disk. The purcell factor is calculated at the position marked by the red point in Figure 4b. As Figure 4c shows, the enhancement of PF in the spectral range matches the resonant modes of LSP and PSP, where PF is enhanced by around 70-fold and 20-fold for LSP and PSP modes, respectively (with the dipole oriented in the OP direction). For comparison, PF with IP dipole is also calculated (red curves), which shows a significant reduction due to the negligible fraction of IP electric fields in these two modes (as clearly shown in Figure 2d,f). As a result, the PLoM structure proposed in this work shows a distinct selective enhancement of the OP and IP dipole emissions.

The lifetime of HS (as shown in Figure 3a,b) was measured by a home-built time-resolved PL (TRPL) system. The radiative lifetime of HS^41−43^ is shortened from *t*_sp0_ ≈1156 ps to *t*_sp_ ≈380 ps (based on the biexponential fitting) after the disk arrays were transferred to the HS structure (Figure 4d), i.e., (1/*t*_sp_)/(1/*t*_sp0_) ≈ 3. The limited spontaneous decay rate enhancement arises from the TRPL signal collected from both the disk-coupled region and the uncoupled region due to the larger laser spot size compared to the disk diameter. Figure S9 shows the PF distribution for a single unit cell (500 nm × 500 nm), where PF is majorly enhanced around the disk. The average Purcell factor, defined as ⟨PF⟩ = ∫PF d*s*/*A*, where *A* is the integral area, is calculated. By taking the integral across the whole unit cell, it will yield a value of 5.8, close to the measured result. If the integral area is shrunk to the disk region, where the field is greatly confined (indicated by the yellow box in Figure S9), ⟨PF⟩ = 47.5 is calculated. As a result, the average enhancement factor within the disk region can be estimated as , which is consistent with our experimental result (∼350) based on eq 1.

Last but not least, the influence of LSP resonance on IX emission is investigated. According to the general formula of the Purcell factor
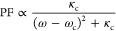
5where ω is the emitter transition frequency, and *k*_c_ and ω_c_ are the decay rate and resonant frequency of the cavity mode, i.e., the LSP mode we discuss here, respectively. To study the effects of LSP resonance on the IX emission, we transferred various disk arrays onto the HS structures.

**Figure 5:**
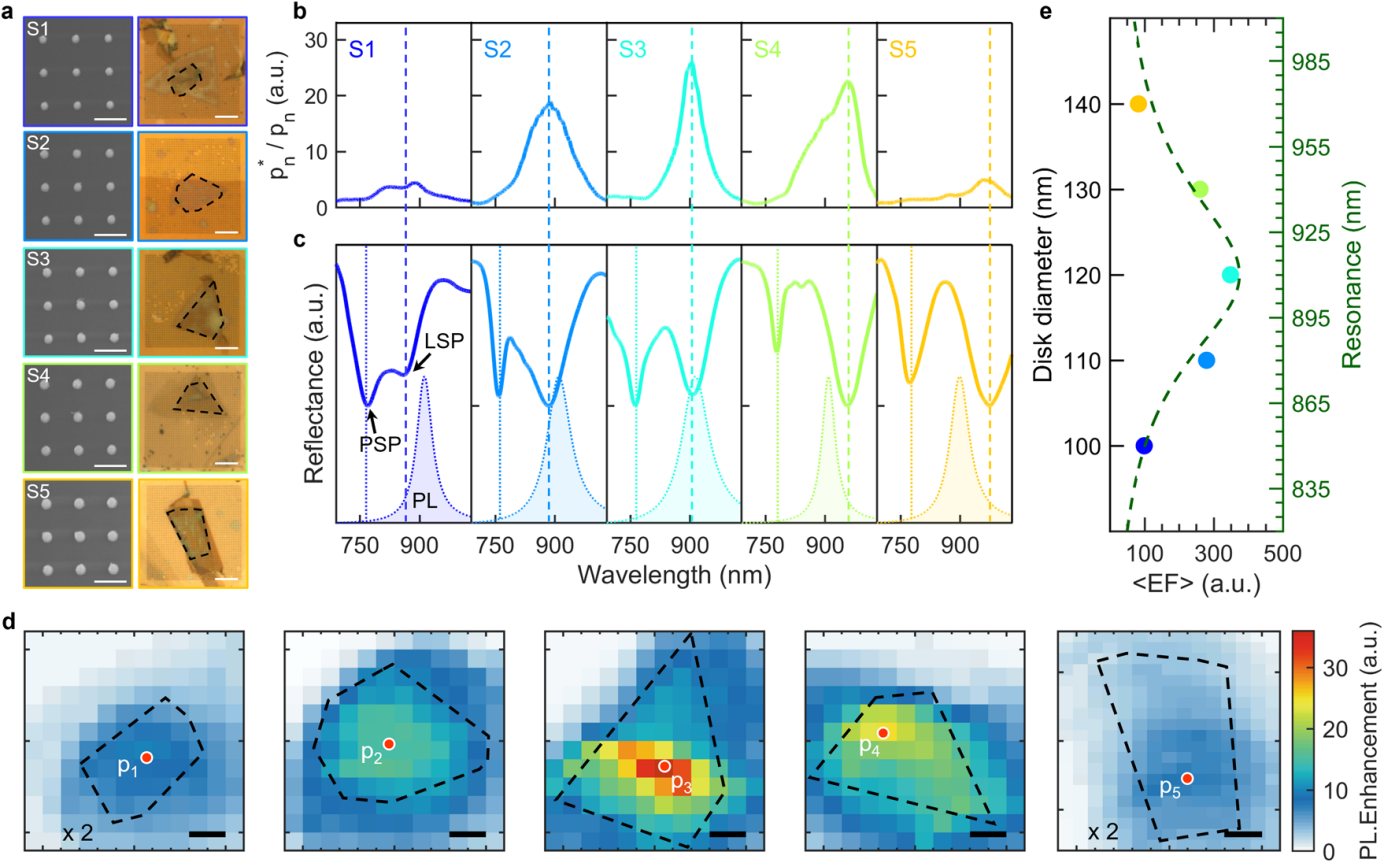
Influence of LSP resonance detuning on IX emission enhancement. (a) Left panel: SEM images (scale bar: 500 nm) of the plasmonic disk array with increasing diameters. Right panel: Corresponding micrographs of HS samples with disk arrays on top (scale bar: 5 μm). The black boxes indicate the HS areas. (b) PL enhancement for five devices. (c) IX (without disk) and reflectance spectra for S1–S5. (d) PL mapping of the devices in Figure 5a. The PL mapping (w/disk) of each device is divided by the mean value of the PL mapping of the corresponding device without a disk (scale bar: 2 μm). The location with the maximum PL intensity is chosen for each device (i.e., p_1_ to p_5_). (e) Colored scatters: calculated ⟨EF⟩ by eq 1 for S1–S5. Dashed line: fitting curve by eq 5.

SEM images in the left panel of Figure 5a display disk arrays with the same pitches (500 nm) but varying diameters (approximately 100, 110, 120, 130, and 140 nm, labeled as S1–S5, respectively). To ensure the similar emission properties of IX, five HS samples were all stacked with 2L-WS_2_ and 5L-InSe, and the total thicknesses of top and bottom BNs are all within the range of 25 to 30 nm (Figure S10). The normalized IX PL of these five HS samples are exhibited in the filled spectra in Figure 5c, all showing resonance peaks at around 905 nm (see temperature evolution of the PL spectra of HS in Figure S11).

After the transfer process, as the right panel of Figure 5a shows, different disk arrays are well retained on the surface of HS samples. The reflectance spectra of these five PLoM structures are measured in the HS region, as indicated in Figure 5c. The PSP mode shows a negligible change when the disk diameter is increased, while the LSP resonance exhibits an unambiguously red shift from 860 to 970 nm. Figure 5b shows the PL enhancement of five samples (S1–S5) by calculating p_n_^*^/p_*n*_ with *n* from 1 to 5 (the locations are indicated in Figure 5d). p_n_^*^ (p_*n*_) represents the PL spectrum measured from the same location as the corresponding HS when HS is coupled (uncoupled) to the nanodisks. In Figure 5b, the PL enhancement shows a pronounced increase from S1 to S3 and then decreases from S3 to S5. In addition, the PL mappings of these five samples are shown in Figure 5d, exhibiting the same tendency as indicated in Figure 5b, i.e., from S1 to S3, the PL mapping increases while decreasing from S3 to S5. The comparison of PL mappings of S1–S5 with and without disk arrays is shown in Figure S12. The modulation of PL enhancement of the HS is ascribed to the variation of LSP resonance when the diameter is increased from 100 to 150 nm (S1 to S5). The distribution of the calculated enhanced factor ⟨EF⟩ with different disk diameters (corresponding to variations of LSP resonances as shown in the right *x*-axis) is exhibited in Figure 5e (colored scatters), which can be well described by the fitting curve (dashed line) calculated by eq 5.

## Conclusions

In conclusion, we have demonstrated a strong modification of the IX peak wavelengths and intensity by tuning the plasmonic nanocavity resonance across the emission spectrum of IX. We obtain an ⟨EF⟩ larger than 350 and attribute the pronounced enhancement to the enhanced spontaneous emission and modified far-field radiation by the nanoantenna. The measured results can be illustrated well by the simulation. Our results reveal a stable and effective areal photoluminescence enhancement approach for IX. The PLoM structure is promising to facilitate OP-dipole-based on-chip optoelectronic devices such as radially polarized sources, nanoantennas, light-emitting diodes, and detectors.^35,37,44−46^

## Experimental Section

### Device Fabrication

A 50 nm gold film was deposited onto a silicon substrate using electron beam evaporation at a deposition rate of 1 Å/s with 5 nm of titanium as an adhesion layer. Bilayer WS_2_, multiple layer InSe, and thin h-BN flakes were mechanically exfoliated on polydimethylsiloxane (PDMS) substrates on a silicon substrate. The heterostructures were stacked using a polypropylene carbonate (PPC) stamp-based alignment transfer technique. The stamp was then dissolved with acetone to release heterostructures onto the gold mirror. The gold nanodisk arrays were fabricated on a 275 nm SiO_2_ Si substrate by electron beam lithography (Elionix ELS7800).

### Morphology Characterization

The atomic force microscope (Dimension Icon, Bruker) was used to check the thickness of h-BN. The morphology of nanodisk arrays was characterized by scan electron microscopy (JEOL7401-F at 5 kV).

### Optical Measurements

All of the optical measurements were performed using a bright-field/dark-field confocal microscope. The photoluminescence spectrum was measured by using aa HR550 Horiba Jobin Yvon spectrometer. PL spectra were measured in an optical cryostat (Linkam) excited at 532 nm laser using a 50× objective with a numerical aperture of 0.5. The laser spot size for excitation is estimated as 1.45 μm (see Figure S14), larger than the moving step (1 μm) for PL mapping. TRPL: A second harmonic 1064 nm (532 nm) Ti: sapphire femtosecond laser beam (Coherent Chameleon Ultra II) with a repetition rate of 80 MHz was used to illuminate the sample, and the signal was collected by a 50× objective (LCPLAN N, NA = 0.65, OLYMPUS, JAPAN). The final signal was then sent to a single-photon detector (MPD PDM Series), and the output was recorded in the Picoharp300 for time-correlated single photon counting (TCSPC) measurements.

## Supporting Information Available

Temperature-dependent PL spectra of device S3; SEM images for the disk arrays with different sizes; schematic diagram of the wet transfer method; experimental and simulated reflectance spectrum of the disk array on the gold mirror (separated by a 30 nm-thick BN layer); PL enhancement spectra at different points of device S3; diagram showing the whole array and one disk unit; electric field distributions of the PLoM structure in the *x*-*y* and *xz*-planes at the excitation wavelength of 532 nm; Purcell factor (PF) distribution within one unit cell; AFM image and height profile acquired from the samples that show the thickness of h-BN in five samples adopted in our measurements; temperature-dependent PL spectra of devices S1–S5; PL mapping of devices S1–S5; and PL collection efficiency with objective of various NAs (PDF)
