# Supplementary material for: Deterministic Areal Enhancement of Interlayer Exciton Emission by a Plasmonic Lattice on Mirror
Source: ACS Nano. 2024 May 14;18(21):13599–606. doi: 10.1021/acsnano.4c00061 (PMC11140836; doi:10.1021/acsnano.4c00061)
Supplement: Supplementary file 1 — nn4c00061_si_001.pdf [file nn4c00061_si_001.pdf]

Deterministic areal enhancement of interlayer exciton emission by a plasmonic lattice on mirror

Jiasen Zhu[1], Fuhuan Shen[1*], Zefeng Chen[2], Feihong Liu[3], Shuaiyu Jin[3], Dangyuan Lei[3*], Jianbin Xu[1*]

1. Electronic Engineering Department of The Chinese University of Hong Kong, Shatin, Hong Kong SAR.
2. School of Optoelectronic Science and Engineering and Collaborative Innovation Center of Suzhou Nano Science and Technology, Soochow University, Suzhou 215006, China
3. Materials Science and Engineering Department of Hong Kong City University.

*Corresponding Authors. Email: fhshen@cuhk.edu.hk (F. H. Shen), dangylei@cityu.edu.hk (D. Y. Lei), and jbxu@ee.cuhk.edu.hk (J. B. Xu)

Supplementary figures:

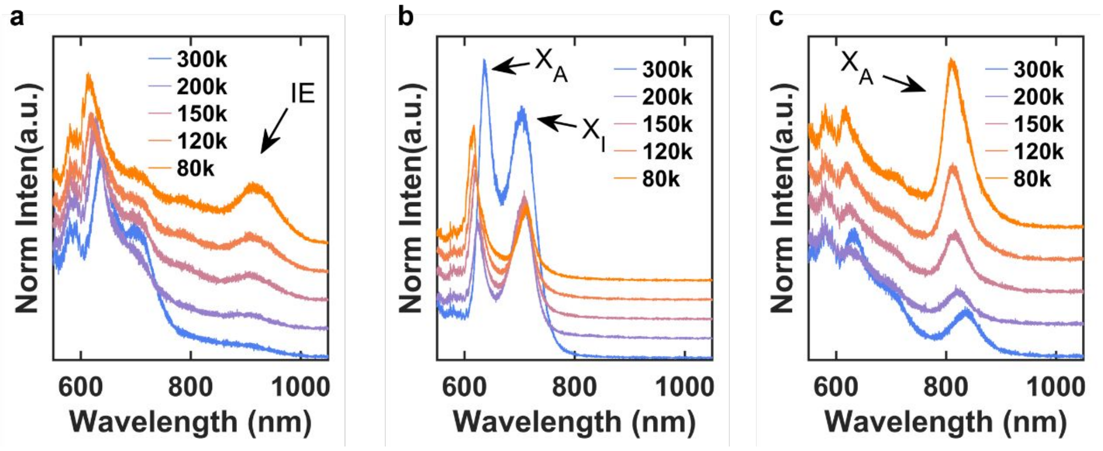

Supplementary Figure 1. Temperature Dependence PL Spectra (a) Temperature-dependent PL spectra of heterostructure (HS). **(b)** Temperature-dependent PL spectra of 2L-WS_2. **(c)** Temperature-dependent PL spectra of InSe.

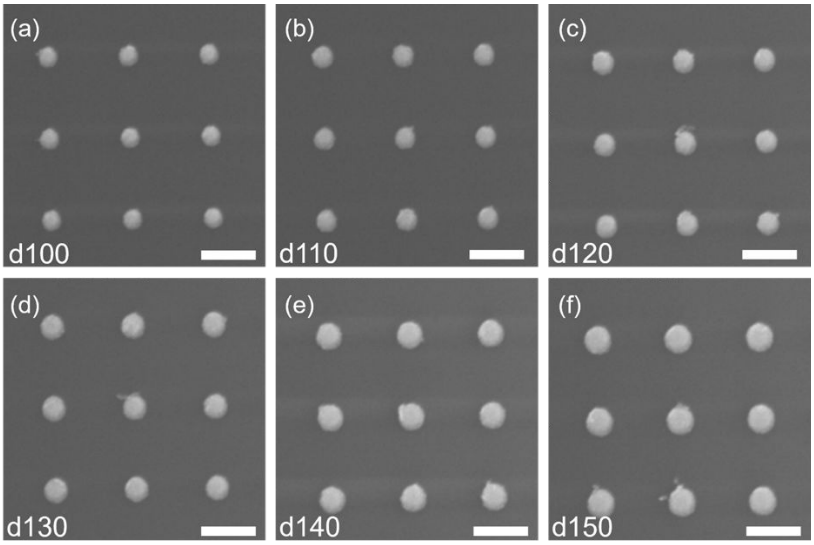

Supplementary Figure 2. Au disk arrays. (a-f) Scanning electron microscope (SEM) image of the disk arrays with disk diameter from 100 nm to 150 nm (labeled from d100 to d150). The period is fixed at 500 nm (scale bar represents 300 nm).

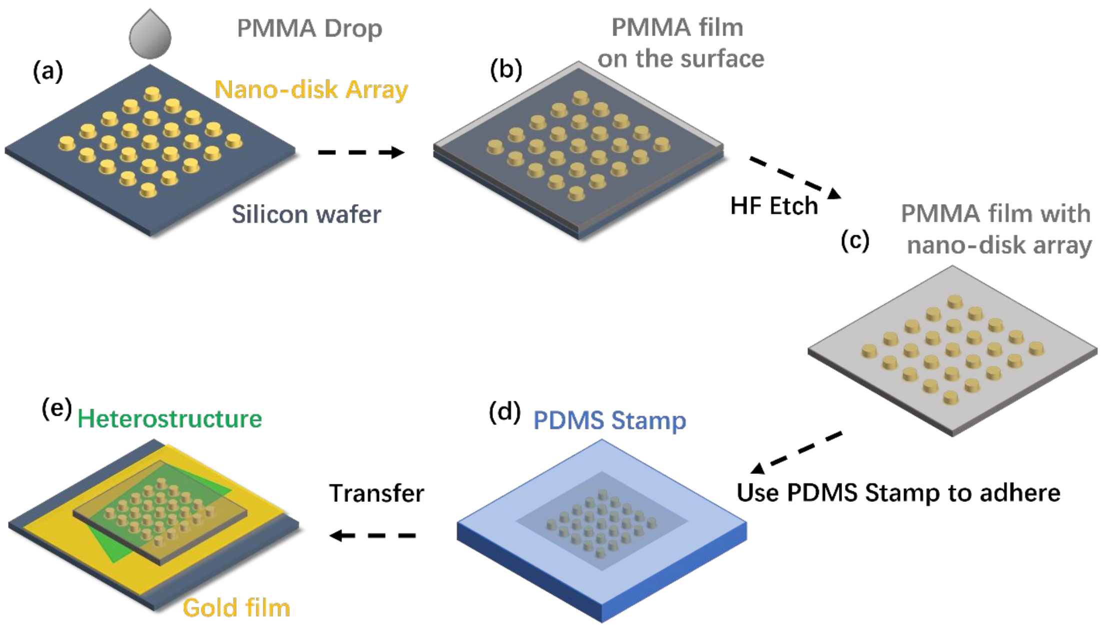

Supplementary Figure 3. Schematic diagram of wet transfer method. (a) Drop PMMA on the substrate with the array. **(b)** Wait for PMMA to form a film and cover the entire substrate. **(c)** Use hydrofluoric acid to etch and get the PMMA film with the array. **(d)** Use PDMS stamp to adhere the PMMA film on glass **(e)** Select the heterostructure to transferred and the PMMA film with the array remains on the area after heating.

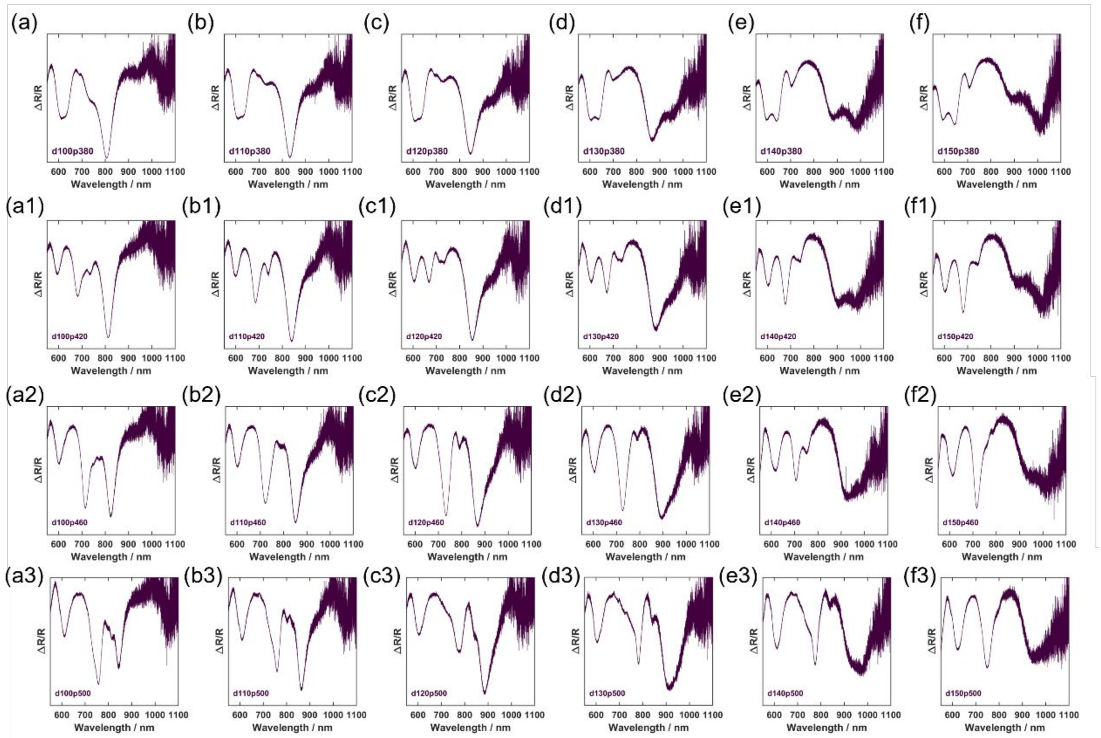

Supplementary Figure 4. Experimental reflectance spectrum of a series of disk array. (a, b, c, d, e, f) Reflectance spectrum of disk array d100p380 to d150p380. **(a1,**

b1, c1, d1, e1, f1) Reflectance spectrum of disk array d100p420 to d150p420. **(a2, b2, c2, d2, e2, f2)** Reflectance spectrum of disk array d100p460 to d150p460. **(a3, b3, c3, d3, e3, f3)** Reflectance spectrum of disk array d100p500 to d150p500. Note that, for instance, d100p500 represent the disk array of diameter 100 nm and period 500 nm.

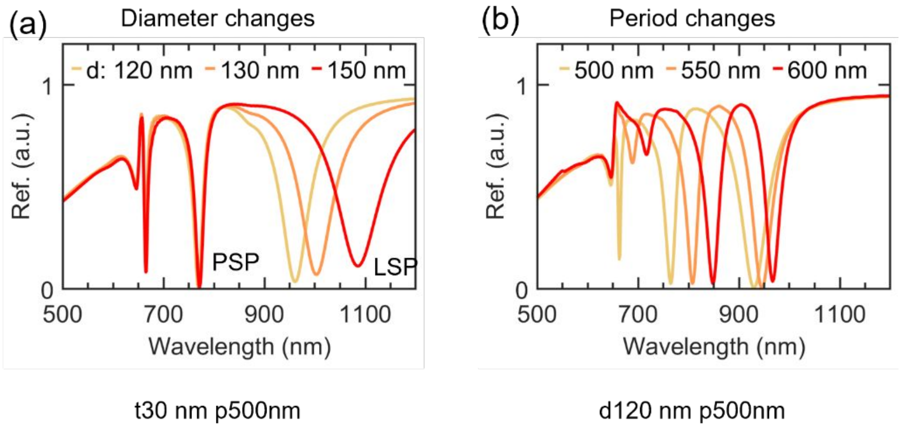

Supplementary Figure 5. Simulated reflectance spectrum of the disk array on 30nm BN on the gold mirror. (a) Simulated reflectance spectrum of the disk array with different diameters. **(b)** Simulated reflectance spectrum of the disk array with different periods.

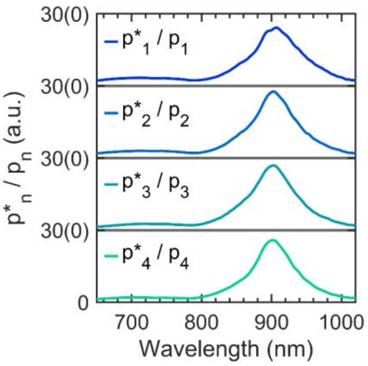

Supplementary Figure 6. PL enhancement spectra at different points in device S3.

Four points from Figure 3c-d in manuscript were adopted for calculation.

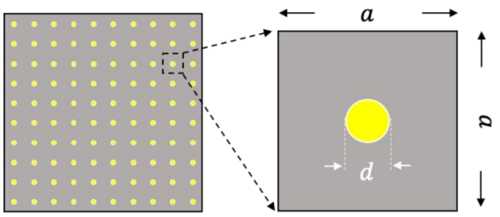

Supplementary Figure 7. Diagram showing the whole array and one disk unit (Enlarged inset).

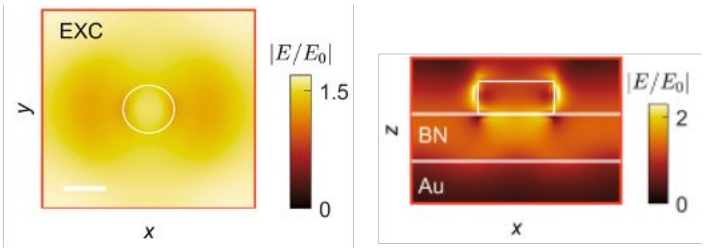

Supplementary Figure 8. Electric field distributions of PLoM structure in x-y and x-z plane at excitation wavelength (532 nm).

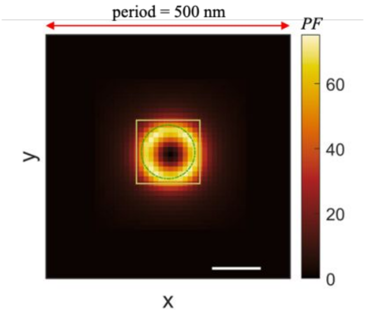

Supplementary Figure 9. Purcell factor (*PF*) field distribution within one disk unit. The period is 500 nm and disk diameter is 110 nm (indicated by the green dashed circle). To calculate the average *PF* around the disk, a yellow square with the side length of 2 × (r + 10 nm) = 130 nm was chosen to count in the Purcell effect due to the near field by the disk edge.

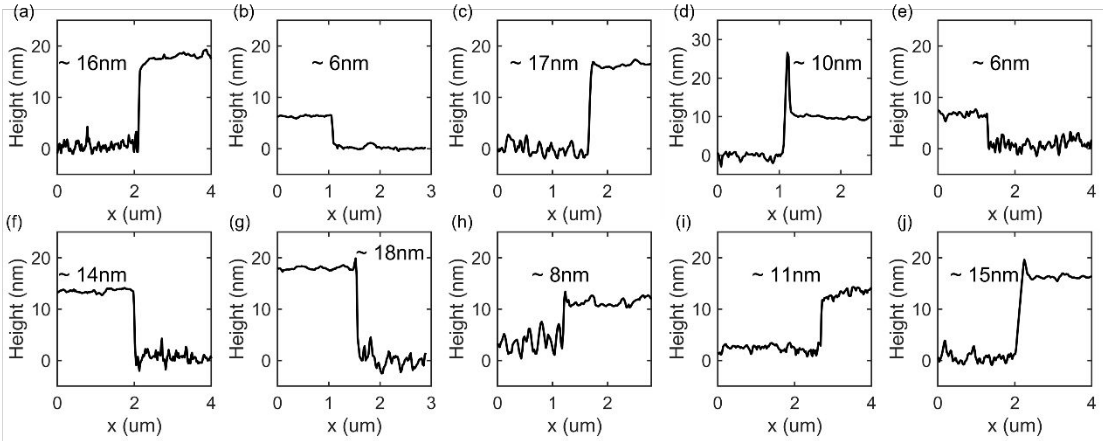

Supplementary Figure 10. Atomic force microscopy of h-BN of Device S1-S5. (a, f) AFM measures the height of top/bottom h-BN of device S1 **(b, g)** AFM measures the height of Top/Bottom h-BN of Device S2 **(c, h)** AFM measures the height of Top/Bottom h-BN of Device S3 **(d, i)** AFM measures the height of top/tottom h-BN of Device S4 **(e, j)** AFM measures the height of top/bottom h-BN of device S5.

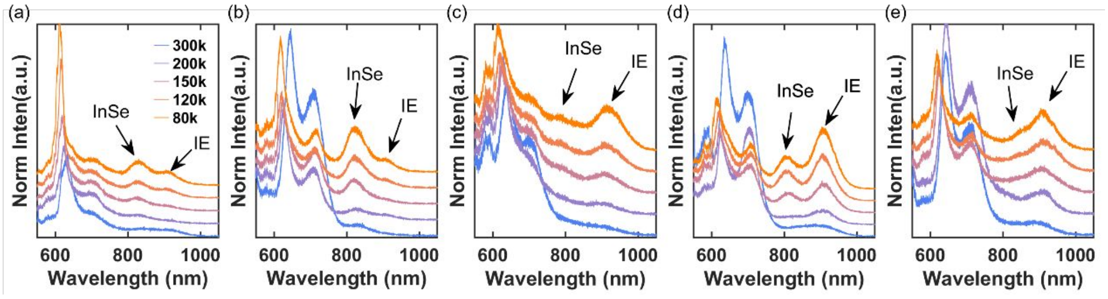

Supplementary Figure 11. Temperature Dependence PL Spectra of Device S1-S5, these spectra share the same legend in Supplementary Figure 2a. (a-e) Temperature Dependence PL Spectra of device S1-S5.

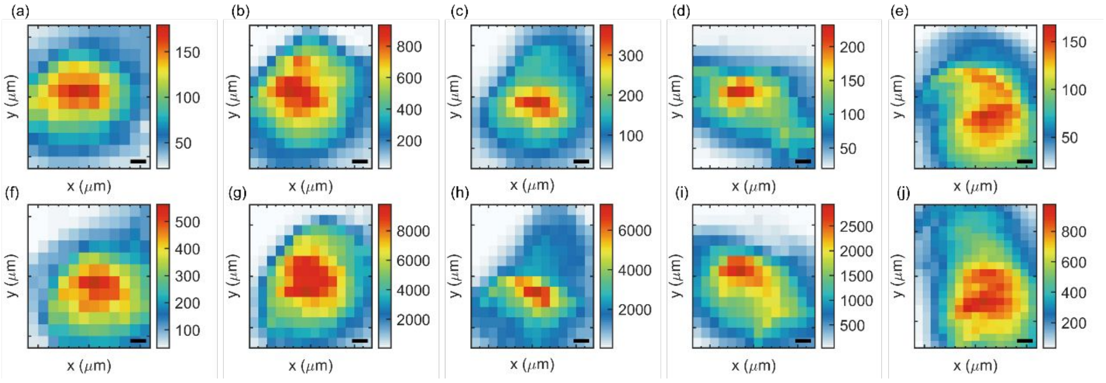

Supplementary Figure 12. PL Mapping of Device S1-S5. (Scale bar: 1.5 um) (a-e) PL mapping (without disk and with disk) of device S1-S5. **(f-j)** PL mapping (with disk) of device S1-S5.

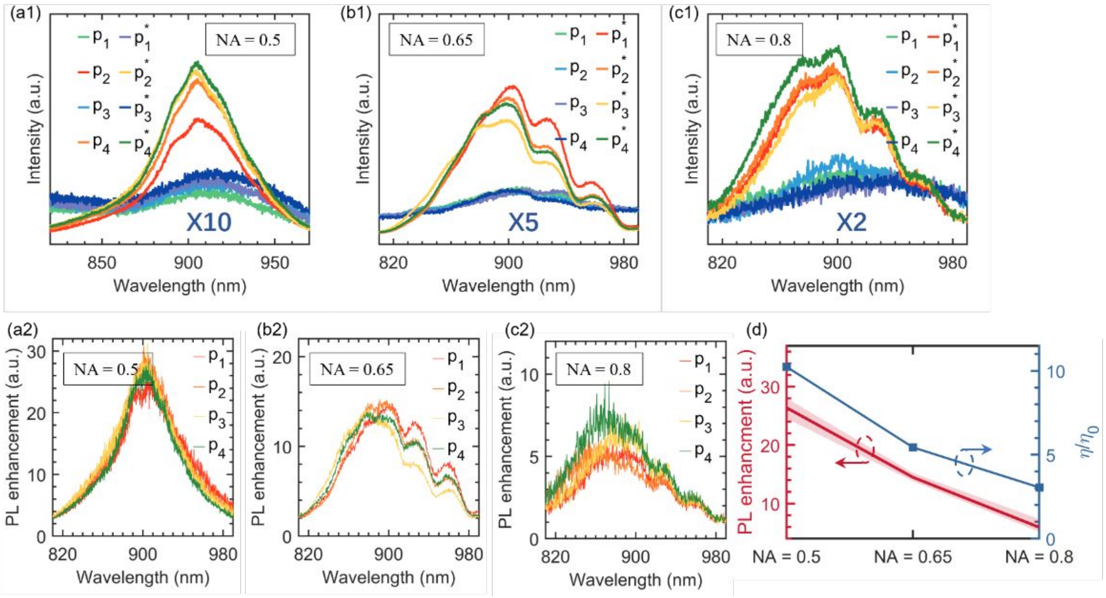

Supplementary Figure 13. PL measurements using objectives with different NAs. PL spectra from four selected locations in HS using objectives with different NA ($p_1 - p_4$represent the locations without disk array while $p_1^* - p_4^*$ represent the

corresponding locations with disk array). **(a1)** NA = 0.5 **(b1)** NA = 0.65 **(c1)** NA = 0.8. Enhancement spectrum at different points using objectives with different NA. **(a2)** NA = 0.5 **(b2)** NA = 0.65 **(c2)** NA = 0.8. **(d)** Collection efficiency & PL Enhancement as a function of objective with different NA. The blue block line shows the simulated collection efficiency as a function of objective with different NA. The red line represents the PL Enhancement as a function of objective with different NA. The shaded area represents the error bar.

We performed PL measurements (Figure-S13) based on objectives with different NA. As shown in Figure-S13-a1; b1; c1, we have measured multiple location in same sample using different objective (NA = 0.5; NA = 0.65; NA = 0.8). When the HS is inserted in the PLoM, IX emission intensities for the same four locations show pronounced enhancements. We calculated the PL enhancements as shown in Figure-S13-a2; b2; c2, the result indicates that the PL enhancement becomes smaller when we use an objective with higher NA. We showed the corresponding enhancement coefficients and error bars as the red line in Figure R2-d and compare it with the calculated collection efficiency as blue line, which showed a high consistency. There are some interference fringes in the PL data of NA = 0.65 and NA = 0.8, but it does not affect our analysis of PL enhancement.

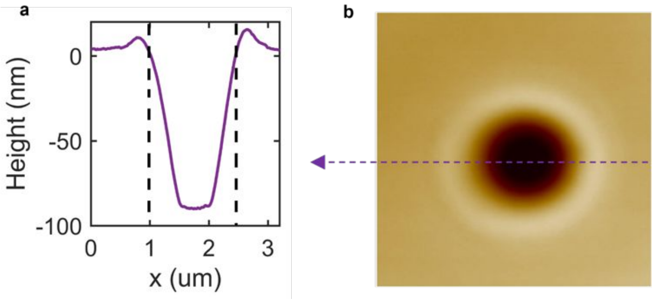

Supplementary Figure 14. Characterization of the PMMA film. (a) Hight profile of the hole left on the PMMA film. (b) Atomic force microscopic (AFM) image of a PMMA film exposed by laser.

Supplementary table:

	NA = 0.5	NA = 0.65	NA = 0.8	NA = 0.9
η	31%	50%	71%	85%
η_0	3%	9%	23%	42%
η/η_0	~10	~5.6	~3	~2

Table S1. **Collection efficiency of objectives with different NAs.**

Collection efficiency: $\eta = \int_{-\arcsin(NA)}^{\arcsin(NA)} \frac{dS^2}{d\Omega^2} \sin\theta d\theta / \int_{-\frac{\pi}{2}}^{\frac{\pi}{2}} \frac{dS^2}{d\Omega^2} \sin\theta d\theta.$
